# Modulation of Arginase-2 mRNA Levels by *ω*-3 PUFAs and Aspirin in Asthmatic Human Lung Fibroblasts

**DOI:** 10.1155/2022/3062274

**Published:** 2022-08-25

**Authors:** Vamsee K. Duggirala, Kyla Geary, Donald Hasenmayer, Farzaneh Daghigh

**Affiliations:** Department of Bio-Medical Sciences, Philadelphia College of Osteopathic Medicine Philadelphia, PA, USA

## Abstract

Airway remodeling (AR) increases disease severity, and morbidity of asthmatic patients by contributing to irreversible airflow obstruction and progressive declines in lung function. Arginase isoenzymes and the downstream enzymes ornithine decarboxylase (ODC) and ornithine aminotransferase (OAT) have been implicated in the hyperplastic and fibrotic changes of AR, respectively. Omega-3 polyunsaturated fatty acids (*ω*-3 PUFAs) and resolvin metabolites have anti-AR effects, but whether they are mediated through the arginase pathway is unclear. Our study intended to determine the effects of the *ω*-3 PUFAs eicosapentaenoic acid (EPA), docosahexaenoic acid (DHA), resolvin D1 (RvD1), T_H_1 cytokines, acetylsalicylic acid (ASA), cAMP, and dexamethasone (DEX) on the expression of arginase isoenzymes arginase 1 (ARG1) and arginase 2 (ARG2), ODC, and OAT in human lung fibroblasts (HLF) from normal (NHLF) and diseased (DHLF) asthmatic donors using reverse transcription-quantitative real-time polymerase chain reaction (RT-qPCR). Our data showed that EPA and EPA+DHA downregulated ARG2 mRNA 2-fold in both types of HLF. DHA, RvD1, and DEX did not alter mRNA levels for any of the genes studied. EPA lowered the ARG2 protein levels in DHLF, but did not affect those levels in NHLF. ASA upregulated ARG2 mRNA 5-fold and 7-fold in NHLF and DHLF, respectively, T_H_1 cytokines downregulated ARG2, ODC, and OAT mRNA in DHLF 10-fold, 2-fold, and 2.5-fold, respectively, and cAMP downregulated ARG2 mRNA 2-fold in DHLF. These results are the first to show a direct effect of *ω*-3 PUFAs on ARG2 mRNA levels and provide further evidence for a role of *ω*-3 PUFAs in AR.

## 1. Introduction

Airway remodeling (AR) in asthma is characterized by increased airway smooth muscle mass, subepithelial fibrosis, epithelial changes, and increased numbers of fibroblasts and myofibroblasts [1]. Established AR is resistant to current asthma therapies and can lead to irreversible airflow limitation, thereby increasing the severity of asthma [2].

Dysregulation in the enzymatic pathways of arginine metabolism, particularly in the arginase pathway, has been shown to contribute to AR [3]. There are two isoforms of arginase, which hydrolyzes arginine to ornithine and urea [4].

Arginase-1 (ARG1) is a liver-type enzyme that functions mainly in the urea cycle, while arginase-2 (ARG2) is expressed in a number of tissues [4]. It has been shown that both ARG1 and ARG2 are expressed in the airways and are upregulated in animal models of asthma as well as in patients [5]. Ornithine decarboxylase (ODC) catalyzes the decarboxylation of ornithine to putrescine, the rate-limiting first step in the synthesis of polyamines, which stimulate cell proliferation [6]. Increased cell proliferation contributes to AR features such as epithelial hyperplasia and increased airway smooth muscle mass [7]. Increased polyamine levels have been detected in patients [8] and animal models of asthma [3, 5]. Ornithine aminotransferase (OAT) converts ornithine to pyrroline-5-carboxylate, an intermediate in the synthesis of proline, an important amino acid in collagen synthesis [4]. This pathway may contribute to subepithelial fibrosis since inhibition of arginase activity reduced collagen synthesis in primary mouse lung fibroblasts induced by transforming growth factor-beta 1 (TGF-*β*_1_) [9].

Studies of the fat-1 transgenic mouse model [10] and paraquat-induced lung fibrosis [11] provide evidence that omega-3 polyunsaturated fatty acids (*ω*-3 PUFAs) and resolvins, their downstream metabolites, may affect AR in addition to their anti-inflammatory effects. The fat-1 gene encodes a desaturase enzyme from *Caenorhabditis elegans* that allows for endogenous production of *ω*-3 PUFAs [10]. Histological analysis comparing the response to allergen challenge following allergic sensitization with aerosolized ovalbumin in both wild-type and fat-1 transgenic mice showed reduced reactive epithelium, leukocyte infiltration, and mucus metaplasia in fat-1 mice compared to wild-type mice [10]. The *ω*-3 PUFA docosahexaenoic acid (DHA) was shown to inhibit the profibrotic changes that occur in paraquat poisoning in Wistar rats, including increased collagen deposition, hydroxyproline content, and upregulated TGF-*β*_1_ mRNA levels [11].

Increasing the ratio of anti-inflammatory *ω*-3 PUFAs to proinflammatory omega-6 polyunsaturated fatty acids (*ω*-6 PUFAs) may reduce AR through modulation of the arginase pathway, since *ω*-6 PUFA-derived prostaglandin E (PGE)1 and PGE2 upregulated arginase activity in RAW 264.7 macrophages significantly more than the *ω*-3 PUFA-derived PGE3 [12].

Although the effects of *ω*-3 PUFAs and resolvins in inflammation have been examined, the direct effects of these compounds on the pathways of arginine metabolism, particularly the arginase pathway, have not been studied in detail. Since the arginase pathway is involved in asthma pathophysiology, it is important to determine the effects of *ω*-3 PUFAs on this pathway in order to find more treatments that target both inflammation and AR in asthma. The present study determined the effects of eicosapentaenoic acid (EPA), DHA, resolvin D1 (RvD1), acetylsalicylic acid (ASA), T_H_1 cytokines, cAMP, and dexamethasone (DEX) on expression of arginase isoenzymes ARG1 and ARG2, ODC, and OAT in human lung fibroblasts (HLF) from normal (NHLF) and diseased (DHLF) asthmatic donors using reverse transcriptase-quantitative real-time polymerase chain reaction (RT-qPCR). ASA, or aspirin, is a known trigger of symptoms in patients with aspirin-intolerant asthma, and given that upregulation of arginase may play a role in asthma pathophysiology, we hypothesized that ASA would upregulate transcription of genes involved in arginine metabolism, while *ω*-3 PUFAs and RvD1 would downregulate transcription of these genes. Although asthma involves a complex interaction between multiple T-helper cytokine subsets, T_H_1 cytokines can inhibit many aspects of T_H_2 cytokine-mediated allergic asthma such as eosinophilia, mucus production, and airway hyperresponsiveness (AHR) [13]. With regard to the arginase pathway, in RAW 264.7 macrophages, upregulation of ARG2 mRNA levels in response to lipopolysaccharide treatment was inhibited by interferon-gamma (IFN-*γ*), but was enhanced by dibutyryl cAMP and DEX [14]. Therefore, we hypothesized that T_H_1 cytokines would downregulate arginase transcription, while cAMP and DEX would upregulate arginase transcription in HLF.

## 2. Materials and Methods

### 2.1. Fibroblast Culture

Two types of HLF were used in this study. NHLF and DHLF, both of which were derived from single donors, were obtained from Lonza (Walkersville, MD). HLF were cultured in Dulbecco's Modification of Eagle's Medium (DMEM) (Cellgro, Mediatech, Inc., Herndon, VA) supplemented with 10% fetal bovine serum (FBS) (Atlanta Biologicals, Lawrenceville, GA) and 1% Antibiotic-Antimycotic (Gibco, Invitrogen Corporation, Life Technologies, Carlsbad, CA) at 37°C in a 5% CO_2_ humidified incubator. HLF between passages 3 and 8 were used for all experiments.

### 2.2. Fibroblast Stimulations

To determine the acute differential effects of *ω*-3 PUFAs on arginine metabolism, HLF were incubated with 200 *μ*M EPA, 200 *μ*M DHA, and a combination of both *ω*-3 PUFAs (100 *μ*M EPA + 100 *μ*M DHA) for 24 hours. EPA sodium salt and DHA sodium salt were purchased from Sigma Aldrich (St. Louis, MO), dissolved in FBS to make 50 mM stock solutions and were stored at -80°C. EPA and DHA stock solutions were freshly diluted in supplemented DMEM prior to treatments. 0.5 mM N-acetylcysteine (NAC) (Sigma Aldrich, St. Louis, MO) was added to all *ω*-3 PUFA treatments to limit peroxidation of *ω*-3 PUFAs in culture. NAC stock solution was prepared by dissolving in phosphate-buffered saline (PBS) (Cellgro, Mediatech, Inc., Herndon, VA) to make a 600 mM stock solution and stored at -20°C.

To determine the acute effects of RvD1 on arginine metabolism, HLF were treated with 1 *μ*M RvD1 (Cayman Chemical, Ann Arbor, MI) for 24 hours alone and in combination with either 10 mM ASA (Sigma Aldrich, St. Louis, MO) or a T_H_1 cytokine mix consisting of 25 ng/mL tumor necrosis factor-alpha (TNF-*α*) (Sigma Aldrich, St. Louis, MO), 10 ng/mL IFN-*γ* (EMD Millipore, Billerica, MA), and 100 ng/mL interleukin-1 beta (IL-1*β*) (Sigma Aldrich, St. Louis, MO). Finally, to determine the acute effects of cAMP and DEX, HLF were treated with 100 *μ*M 8-bromo-cAMP (8-Br-cAMP) (Calbiochem, EMD Millipore, Billerica, MA), which is a cAMP analog resistant to degradation by phosphodiesterases, 2 *μ*g/mL DEX (Sigma Aldrich, St. Louis, MO), or both for 24 hours. The concentrations used for all stimulations were determined through dose-response experiments (data not shown).

### 2.3. Reverse Transcriptase-Quantitative Real-Time Polymerase Chain Reaction (RT-qPCR)

RNA was extracted from HLF using the RNeasy Plus Micro kit (Qiagen, Valencia, CA) and quantified using the NanoDrop 2000c UV-Vis Spectrophotometer (Thermo Scientific, USA). 5 *μ*g of RNA was used to synthesize complementary DNA (cDNA) using the Superscript II Reverse Transcriptase kit (Invitrogen, Life Technologies, Carlsbad, CA) according to the manufacturer's instructions for cDNA synthesis using random primers.

Random-9-mer primers were purchased from Agilent Technologies (New Castle, DE). cDNA was diluted 5x prior to qPCR. qPCR was conducted using the Premix Ex Taq Perfect Real-Time kit (Clontech Laboratories, Takara Bio, Mountain View, CA) and Gene Expression Assay hydrolysis probes (Applied Biosystems, Life Technologies, Carlsbad, CA) for glyceraldehyde 3-phosphate dehydrogenase (GAPDH) (Hs02758991_g1), ARG1 (Hs00968979_m1), ARG2 (Hs00982833_m1), ODC (Hs00159739_m1), and OAT (Hs00236852_m1). Reactions were performed in triplicate using the ABI Prism 7000 Sequence Detection System under the following thermal cycling conditions: initial denaturation at 95°C for 2 minutes followed by 40 cycles of 95°C for 5 seconds and 60°C for 37 seconds. Data were normalized using the reference gene GAPDH. The comparative quantification cycle (*ΔΔ*Cq) method was used to determine fold-change in mRNA levels relative to untreated control samples.

### 2.4. Enzyme-Linked Immunosorbent Assay (ELISA)

The Arginase Liver Type Human ELISA kit (Biovendor, Ashville, NC) was used to determine ARG1 protein levels in HLF whole cell lysates according to the manufacturer's instructions. The ELISA Kit for ARG2 (http://MyBioSource.com) was used to detect ARG2 levels using 50 *μ*g of mitochondrial, HLF protein fractions according to manufacturer's instructions.

### 2.5. Statistical Analysis

Results are presented as mean relative mRNA levels ± standard error of the mean (SEM) for at least *n* = 3 biological replicates performed in triplicate. Since each biological replicate consisted of all treatments, a randomized block ANOVA followed by Tukey's multiple comparisons test was used to determine significant differences between treatment groups, with biological replicate as the blocking variable. Graphpad Prism 6 statistical software package (Graphpad Software, San Diego, USA) was used to perform all statistical analyses. A *P* value less than 0.05 was considered statistically significant.

## 3. Results

### 3.1. Basal Expression Levels of Arginase Isoenzymes

In both untreated and treated HLF, ARG1 mRNA and protein were undetectable in HLF by RT-PCR and ELISA, respectively. On the other hand, the ARG2 isoenzyme mRNA and protein levels were reliably detectable in both untreated and treated HLF and, therefore, were studied extensively.

### 3.2. Effects of *ω*-3 PUFAs on Transcription of Genes Involved in Arginine Metabolism

**Figure 1:**
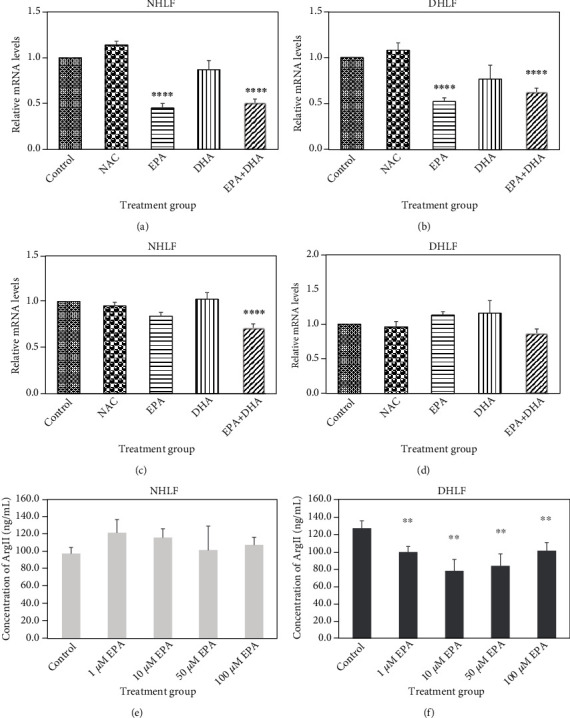
The effects of *ω*-3 PUFAs on ARG2 and ODC mRNA levels and ARG2 protein levels in HLF. ARG2 (a and b) and ODC (c and d) mRNA levels were determined in NHLF (a and c) and DHLF (b and d). HLF were treated with 0.5 mM NAC in combination with either 200 *μ*M EPA, 200 *μ*M DHA, or EPA+DHA (100 *μ*M each) for 24 hours. RNA was then extracted from HLF and analyzed by RT-qPCR. ARG2 (e and f) protein levels were determined utilizing ELISA, HLF cells were treated with 0 (Control), 1, 10, 50, and 100 *μ*M for 24 hours. Data were statistically analyzed using randomized block ANOVA, followed by Tukey's multiple comparisons test. Depicted are the mean relative mRNA levels ± SEM for *n* = 5 biological replicates performed in triplicate. Data from ELISA experiments (*n* = 4) are displayed as the mean protein concentrations ± SEM. Stars indicate significant differences determined using Tukey's multiple comparisons test as follows: ^∗^*P* < 0.05, ^∗∗^*P* < 0.001, and ^∗∗∗^*P* < 0.0001 significant difference from control.

Experiments with *ω*-3 PUFAs showed that EPA reduced ARG2 mRNA levels by half relative to untreated control, which corresponds to a 2-fold downregulation in ARG2 mRNA levels in both NHLF (Figure 1(a), *P* < 0.0001) and DHLF (Figure 1(b), *P* < 0.0001). EPA+DHA also downregulated ARG2 mRNA levels approximately 2-fold relative to untreated control in NHLF (Figure 1(a), *P* < 0.0001), and in DHLF, the combination treatment downregulated ARG2 mRNA levels approximately 1.7-fold (Figure 1(b), *P* < 0.001). In both types of HLF, there was no significant difference between the EPA treatment and EPA+DHA treatment. EPA+DHA also downregulated ODC mRNA levels in NHLF 1.4-fold (Figure 1(c), *P* < 0.0001).

ARG2 protein levels were significantly lowered when treated with 1, 10, 50, and 100 *μ*g relative to untreated control (Figure 1(e), *P* < 0.05). NAC and DHA treatments did not significantly alter ARG2 and ODC mRNA levels. OAT mRNA levels did not change significantly in response to NAC or any of the *ω*-3 PUFA treatments (data not shown).

### 3.3. Effects of EPA on ARG2 Protein Levels

The expression levels of ARG2 isoenzyme in absence and presence of EPA (1, 10, 50, and 100 *μ*M) were detected in NHLF or DHLF via ELISA in treated HLF cells. EPA lowered the ARG2 protein expression significantly (Figure 1(b), *P* < 0.05) even though it did not affect the protein levels of NHLF.

### 3.4. Effects of RvD1 in the Presence of T_H_1 Cytokines and ASA on Transcription of Genes Involved in Arginine Metabolism

**Figure 2:**
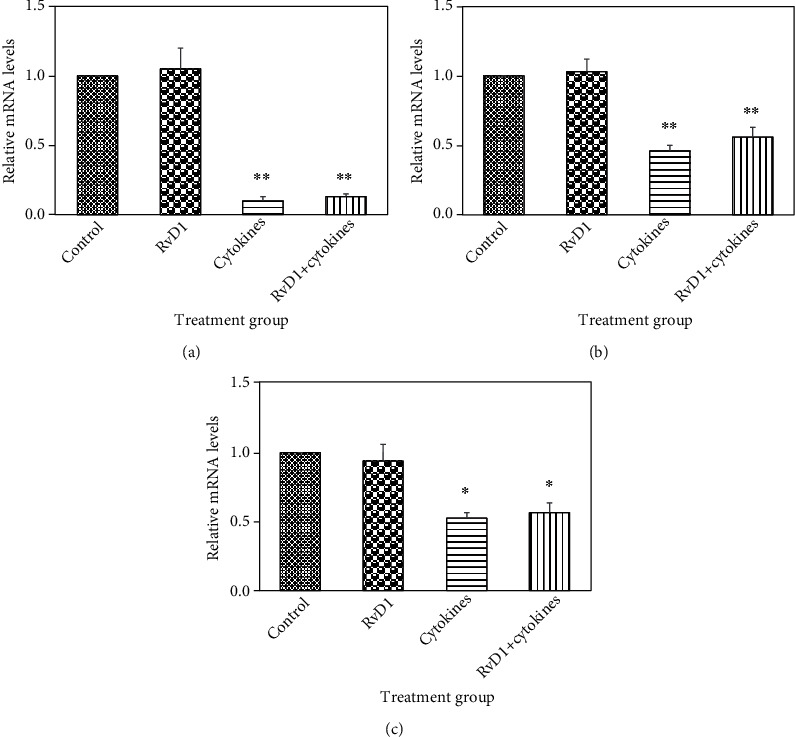
The effects of RvD1 and T_H_1 cytokines on mRNA levels of genes involved in arginine metabolism in DHLF. ARG2 (a), ODC (b), and OAT (c) mRNA levels were determined in DHLF. DHLF were treated with 1 *μ*M RvD1, T_H_1 cytokine cocktail (25 ng/mL TNF-*α*, 10 ng/mL IFN-*γ*, and 100 ng/mL IL- 1*β*), or both for 24 hours. RNA was then extracted from DHLF and analyzed by RT-qPCR. Data were statistically analyzed using randomized block ANOVA, followed by Tukey's multiple comparisons test. Depicted are the mean relative mRNA levels ± SEM for *n* = 3 biological replicates performed in triplicate. Stars indicate significant differences determined using Tukey's multiple comparisons test as follows: ^∗^*P* < 0.05 and ^∗∗^*P* < 0.01 significant difference from control.

**Figure 3:**
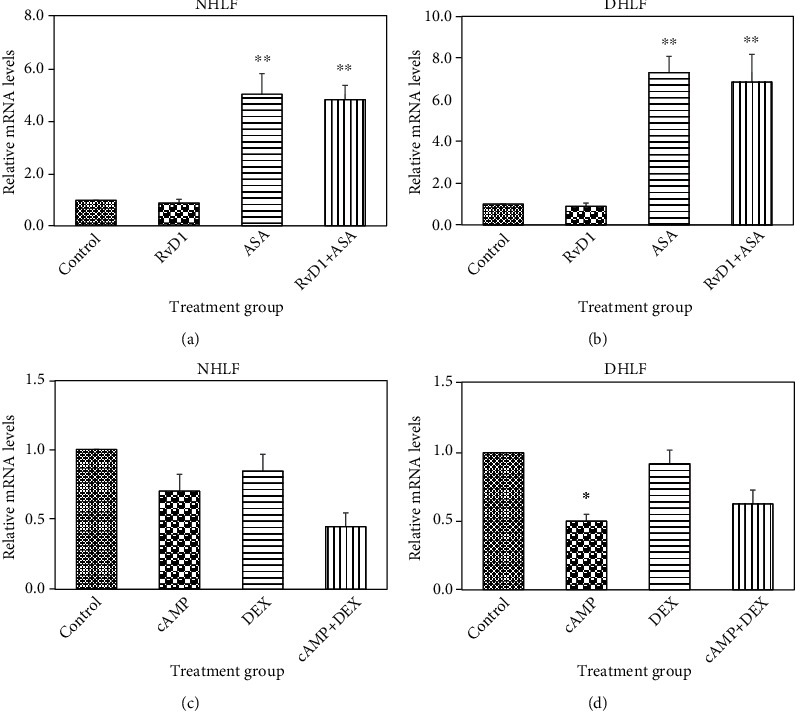
The effects of RvD1, ASA, cAMP, and DEX on ARG2 mRNA levels in HLF. ARG2 mRNA levels were assessed in NHLF (a and c) and DHLF (b and d). HLF were treated with 1 *μ*M RvD1, 10 mM ASA, 100 *μ*M 8-Br-cAMP, or 2 *μ*g/mL DEX for 24 hours. RNA was then extracted from HLF and analyzed by RT-qPCR. Data were statistically analyzed using randomized block ANOVA, followed by Tukey's multiple comparisons test. Depicted are the mean relative mRNA levels ± SEM for *n* = 3 biological replicates performed in triplicate for all experiments, except for the cAMP experiment in DHLF (d), for which *n* = 4 biological replicates were performed in triplicate. Stars indicate significant differences determined using Tukey's multiple comparisons test as follows: ^∗^*P* < 0.05 and ^∗∗^*P* < 0.01 significant difference from control.

RvD1 did not significantly modify mRNA levels for ARG2, ODC, and OAT in either type of HLF. This is consistent with the results of *ω*-3 PUFA experiments in which DHA, which is metabolized to RvD1, also did not affect mRNA levels of any of the genes of interest. T_H_1 cytokines downregulated ARG2 mRNA levels 10-fold in DHLF (Figure 2(a), *P* < 0.01). T_H_1 cytokines also downregulated ODC mRNA levels 1.7-fold (Figure 2(b), *P* < 0.01) and OAT mRNA levels 2-fold (Figure 2(c), *P* < 0.05) in DHLF. T_H_1 cytokines did not alter mRNA levels of ARG2, ODC, and OAT in NHLF (data not shown). ASA upregulated ARG2 mRNA levels in NHLF and DHLF 5-fold (Figure 3(a), *P* < 0.01) and 7-fold (Figure 3(b), *P* < 0.01) relative to untreated control, respectively.

### 3.5. Effects of cAMP and DEX on Transcription of Genes Involved in Arginine Metabolism

cAMP downregulated ARG2 mRNA levels 2-fold in DHLF (*P* < 0.05) (Figure 3(d)). cAMP+DEX downregulated ARG2 mRNA levels 2-fold in NHLF, but this decrease did not reach statistical significance (Figure 3(c)). None of the treatments involving cAMP and DEX significantly modulated ODC and OAT mRNA levels in either type of HLF (data not shown).

## 4. Discussion

The results from the current study demonstrate that *ω*-3 PUFAs, T_H_1 cytokines, and cAMP downregulate ARG2 mRNA levels, while ASA upregulates ARG2 mRNA levels; however, these effects were not all the same depending on the type of HLF. *ω*-3 PUFAs and ASA exerted their effects in both types of HLF, whereas cAMP and T_H_1 cytokines only downregulated ARG2 mRNA levels in DHLF. T_H_1 cytokines also downregulated ODC and OAT mRNA levels significantly in DHLF, but not to the same extent as that observed for ARG2 mRNA levels. Similarly, EPA+DHA treatment led to a small but significant downregulation of ODC mRNA levels in NHLF.

The limited modulation observed on ODC and OAT mRNA levels compared to ARG2 mRNA levels may indicate that the synthesis of polyamines and proline is dependent on ARG2 expression and arginine availability rather than ODC and OAT expression. This would be in agreement with a previous study, which demonstrated that in the presence of excess ornithine, proline production in murine bone marrow-derived macrophages stimulated with T_H_2 cytokines increased only 2-fold, whereas in the presence of excess arginine, proline production increased 13-fold [15].

Furthermore, *N*^*ω*^-hydroxy-nor-arginine, an ARG1-specific inhibitor and an intermediate generated during production of nitric oxide by nitric oxide synthase (NOS), abolished the increased production of ornithine and the polyamine spermine in both murine bone marrow-derived macrophages infected with Leishmania major, which elicits a T_H_2 response, and uninfected macrophages induced by IL-4 [16].

The differential expression of arginase isoenzymes in HLF contrasts with previous data that implicates both isoenzymes in asthma and other T_H_2 cytokine-mediated diseases. Zimmerman et al. showed using microarray analysis that in both ovalbumin-sensitized and *Aspergillus fumigatus*-sensitized mice, ARG1 mRNA levels were upregulated 40-fold and 20-fold, respectively, whereas ARG2 mRNA levels were upregulated only 3-fold in both models of allergic asthma [5]. In the mouse model of asthma consisting of NOS2 knockout mice, allergen challenge upregulated protein levels of ARG1 and ARG2 40-fold and 4-fold, respectively [17]. The difference in arginase isoenzyme expression observed in the HLF in the current study and that observed in other studies may involve species-specific differences in arginase isoenzyme expression. Warnken et al. showed using qPCR analysis that in primary rat lung fibroblasts ARG1 was the predominant isoenzyme expressed, but in primary HLF ARG1 was at or below the detection limit. ARG2 mRNA levels, on the other hand, were dramatically greater in HLF than in primary rat lung fibroblasts [18]. The results of the current study agree with those of Warnken et al. in suggesting that ARG2 is the predominant isoenzyme expressed in HLF.

The upregulation of ARG2 mRNA levels in response to ASA treatment and the downregulation in ARG2 mRNA levels observed in response to cAMP treatment may involve blockade of prostaglandin signaling via cAMP. Shi et al. showed that ASA downregulates arginase activity in polymorphonuclear myeloid-derived suppressor cells from ovalbumin-sensitized mice [19]. The mechanism for this downregulation involved blockade of PGE2 synthesis, leading to reduced activation of the E prostanoid receptor 4 (EP4) and downstream signal transduction via the cAMP/protein kinase A (PKA) pathway [19]. Contrary to the finding of Shi et al., in the present study, ASA upregulated ARG2 mRNA levels, while cAMP downregulated ARG2 mRNA levels. This is the opposite of what was observed by Shi et al. and suggests that the effects of PGE2 may be mediated through a different cAMP signal transduction pathway in HLF. Chen et al. demonstrated that cAMP prevents hypoxia-induced upregulation of ARG2 mRNA and protein levels in human pulmonary artery smooth muscle cells via exchange protein directly activated by cAMP (Epac) pathway [20]. PGE2-induced intercellular adhesion molecule-1 expression was shown to be mediated by EP4 signaling via the Epac pathway in brain endothelial cells [21]. Alternatively, PGE3 may be the prostaglandin involved since PGE3 also interacts with EP4 [22], and EPA, the *ω*-3 PUFA that cyclooxygenase metabolizes to PGE3, downregulated ARG2 mRNA levels in HLF in the current study. Together, the upregulation of ARG2 in response to ASA in HLF observed in the current study may be due to blockade of PGE2 and/or PGE3 production, leading to subsequent disruption in signaling through EP4, cAMP, and Epac. The upregulatory effect of ASA on ARG2 mRNA levels points to a novel mechanism for AR in aspirin-intolerant asthma. Patients with this form of asthma show increased disease severity as well as remodeling of the airways that contributes to irreversible airflow obstruction [23]. The effect of ASA demonstrated in the present study warrants further investigation into the role of the arginase pathway in aspirin-intolerant asthma.

With regard to the effect of *ω*-3 PUFAs on the arginase pathway, the downregulatory effect of EPA and EPA+DHA on ARG2 mRNA levels may involve the liver X receptor (LXR), a nuclear receptor that regulates fatty acid and inflammatory gene expression [24]. LXRs exert their effects by forming a heterodimer with the retinoid X receptors (RXR) that binds LXR response elements (LXRRE) in LXR-regulated genes [25].

Marathe et al. determined that the ARG2 gene promotor contains an LXRRE and showed that LXR agonist and RXR agonist both upregulated ARG2 mRNA levels in RAW 264.7 macrophages stably transfected with LXR plasmids as well as in primary mouse peritoneal macrophages, with both agonists together eliciting an additive upregulatory effect on ARG2 mRNA levels [26]. *ω*-3 PUFAs inhibit LXRs through a number of mechanisms, some of which involve competitive inhibition of LXR activation [27], stimulation of peroxisome proliferator-activated receptors that compete with LXR for heterodimer formation with RXR [28], and/or stimulation of farnesol X receptor, which increases expression of short heterodimer protein that exerts a negative feedback effect on LXR activity [29].

## 5. Conclusions

Further confirmation of the changes in mRNA levels through analysis of protein levels and enzymatic activity is required; arginase activity is generally proportional to protein levels, which are determined at the transcriptional level [30]. For example, the histone deacetylase inhibitor trichostatin A upregulated arginase activity in human aortic endothelial cells and mouse aortic rings. Using RNA interference, it was determined that this upregulation of arginase activity was due to upregulation of ARG2 mRNA and protein, both of which resulted from increased ARG2 promoter activity [31]. In another study, hypoxia upregulated arginase activity in human microvascular endothelial cells, and this correlated with upregulation of ARG2 mRNA and protein that was mediated by the transcription factor hypoxia-inducible factor-2 [32]. The results from the current study serve as a starting point to further elucidate the mechanisms by which *ω*-3 PUFAs, T_H_1 cytokines, ASA, and cAMP modulate the arginase pathway in HLF. These findings confirm previous studies pointing to the therapeutic potential of *ω*-3 PUFAs in asthma [33]. Moreover, they provide novel evidence that the arginase pathway is a direct target of *ω*-3 PUFA modulation. These preliminary findings support further research into dietary interventions for asthma using *ω*-3 PUFAs for patients with significant AR.

## Data Availability

The data used to support the findings of this study are included within the article.

## Abbreviations

8-Br-cAMP: 8-Bromo-cAMP
AR: Airway remodeling
ASA: Acetylsalicylic acid
DEX: Dexamethasone
DHLF: Diseased human lung fibroblasts
Epac: Exchange protein directly activated by cAMP
EP4: E prostanoid receptor 4
GAPDH: Glyceraldehyde 3-phosphate dehydrogenase
HLF: Human lung fibroblast
LXR: Liver X receptor
LXRRE: Liver X receptor response element
*ω*-6 PUFA: Omega-6 polyunsaturated fatty acid
*ω*-3 PUFA: Omega-3 polyunsaturated fatty acid
NAC: N-Acetylcysteine
NHLF: Normal human lung fibroblasts
NOS: Nitric oxide synthase
OAT: Ornithine aminotransferase
ODC: Ornithine decarboxylase
PGE: Prostaglandin E
RvD1: Resolvin D1
RT-qPCR: Reverse transcription-quantitative real-time polymerase chain reaction
RXR: Retinoid X receptor
TGF-*β*_1_: Transforming growth factor-beta 1.
